# Differential contribution of canonical and noncanonical NLGN3 pathways to early social development and memory performance

**DOI:** 10.1186/s13041-024-01087-5

**Published:** 2024-03-12

**Authors:** Lin-Yu Li, Ayako Imai, Hironori Izumi, Ran Inoue, Yumie Koshidaka, Keizo Takao, Hisashi Mori, Tomoyuki Yoshida

**Affiliations:** 1 Department of Molecular Neuroscience, Faculty of Medicine, University of Toyama, Toyama, 930-0194 Japan; 2 Research Center for Idling Brain Science, University of Toyama, Toyama, 930-0194 Japan; 3 Division of Experimental Animal Resource and Development, Life Science Research Center, University of Toyama, Toyama, 930-0194 Japan; 4 Department of Behavioral Physiology, Faculty of Medicine, University of Toyama, Toyama, 930-0194 Japan

**Keywords:** Neuroligin 3, Neurexins, Protein tyrosine phosphatase δ, Synapse organizer, Social behavior, Memory

## Abstract

Neuroligin (NLGN) 3 is a postsynaptic cell adhesion protein organizing synapse formation through two different types of transsynaptic interactions, canonical interaction with neurexins (NRXNs) and a recently identified noncanonical interaction with protein tyrosine phosphatase (PTP) δ. Although, *NLGN3* gene is known as a risk gene for neurodevelopmental disorders such as autism spectrum disorder (ASD) and intellectual disability (ID), the pathogenic contribution of the canonical NLGN3-NRXN and noncanonical NLGN3-PTPδ pathways to these disorders remains elusive. In this study, we utilized *Nlgn3* mutant mice selectively lacking the interaction with either NRXNs or PTPδ and investigated their social and memory performance. Neither *Nlgn3* mutants showed any social cognitive deficiency in the social novelty recognition test. However, the *Nlgn3* mutant mice lacking the PTPδ pathway exhibited significant decline in the social conditioned place preference (sCPP) at the juvenile stage, suggesting the involvement of the NLGN3-PTPδ pathway in the regulation of social motivation and reward. In terms of learning and memory, disrupting the canonical NRXN pathway attenuated contextual fear conditioning while disrupting the noncanonical NLGN3-PTPδ pathway enhanced it. Furthermore, disruption of the NLGN3-PTPδ pathway negatively affected the remote spatial reference memory in the Barnes maze test. These findings highlight the differential contributions of the canonical NLGN3-NRXN and noncanonical NLGN3-PTPδ synaptogenic pathways to the regulation of higher order brain functions associated with ASD and ID.

**Supplementary Information:**

The online version contains supplementary material available at 10.1186/s13041-024-01087-5.

## Introduction

Neuroligin (NLGN) 3 is a member of NLGN family consisting of NLGN1, 2, 3, 4X, and 4Y, which share common domain architecture composed of an N-terminal signal sequence, an extracellular cholinesterase-like domain, a single-pass transmembrane segment, and a short cytoplasmic domain [1]. It is well-established that NLGNs at the postsynaptic membrane induce synaptogenesis, stabilize and maintain mature synapses, and regulate synaptic plasticity though interactions with presynaptic neurexins (NRXNs) [2, 3]. *NLGN3* is one of the best-characterized X-linked genes, with various mutations being associated with the pathogenesis of neurodevelopmental disorders such as autism spectrum disorders (ASD) and intellectual disability (ID) [4–7]. ASD is characterized by deficits in social communication and repetitive behaviors, while ID is characterized by significant limitations in intellectual functioning and adaptive behaviors [8]. Among *NLGN3* mutations, the arginine-to-cysteine substitution at the 451st amino acid residue (R451C) in the cholinesterase-like domain was first identified in a Swedish family with ASD [4]. The *Nlgn3* knock-in mice on a B6/129S background carrying the humanized R451C mutation displayed social deficits and enhanced spatial learning [9], while no obvious deficit in social and cognitive functions was observed in the knock-in mice on a pure B6 background [10]. Similarly, *Nlgn3* knockout (KO) mice showed ASD and ID-related behavioral phenotypes including impaired social recognition, social memory, and fear conditioning [11–14]. The phenotypes of these *Nlgn3* mutant mice have long been considered to be due to a lack or change in the transsynaptic interactions between NLGN3 and NRXNs. Recently, we discovered a noncanonical interaction between NLGN3 and protein tyrosine phosphatase (PTP) δ, which competes with the canonical interaction with NRXNs [15]. The identification of the noncanonical transsynaptic pathway raised the question of how these two pathways of NLGN3 contribute to the development of social and cognitive functions linked to ASD and ID symptoms. Surprisingly, the *Nlgn3* mutant mice (*Nlgn3*^*hse*^ line) designed to selectively lack the canonical NRXN-interaction showed increased social interaction in the 3-chamber and reciprocal social interaction tests while *Nlgn3*^*mf*^ mice, with impaired noncanonical PTPδ-interaction, exhibited no social preference and often adopted defensive postures in adulthood [15]. In this study, we utilized these *Nlgn3* mutant mice to examine the effects of impairments in canonical or noncanonical NLGN3 synaptogenic pathways on early social development and the cognitive functions related to ASD and ID pathogenesis. Neither *Nlgn3*^*hse*^ nor *Nlgn3*^*mf*^ mutants showed any obvious social cognitive deficiency in the social novelty test while the *Nlgn3*^*mf*^ mutants exhibited significant decline in the social conditioned place preference (sCPP) at the juvenile stage. In terms of learning and memory, *Nlgn3*^*hse*^ mutant mice exhibited attenuated fear conditioning. In contrast, *Nlgn3*^*mf*^ mutant mice showed increased fear conditioning and memory and a significant impairment in remote spatial reference memory as observed in the Barnes maze test. Our results revealed redundant and differential contributions of the canonical and noncanonical NLGN3 synaptogenic pathways to the development of social and cognitive functions associated with ASD and ID.

## Materials and methods

### Animals

All the procedures were approved by the Animal Experiment Committee of the University of Toyama (Authorization No. A2023MED-05 and A2016OPR-3) and conducted in accordance with the Guidelines for the Care and Use of Laboratory Animals of the University of Toyama. *Nlgn3*^*hse*^ line and *Nlgn3*^*mf*^ line mice with a pure C57BL/6N genetic background had been reported previously [15]. Mice were housed in a room under a 12 h light/dark cycle (lights on at 7:00 a.m.) at 23 ± 3 °C and 30–60% humidity. Food and water were provided ad libitum. Wild-type (WT) and mutant mice were generated by crossing heterozygous female mice with WT male mice. All behavioral analyses were performed on male mice only. For general behavioral testing, the male offspring of mating pairs were weaned around one month, genotyped, and housed 4 (two pairs of WT and mutant mice) per cage. For the five-trial social novelty test, male offspring mice were weaned at postnatal day (P) 21-P23 and randomly assigned 3–5 animals per cage.

### Behavioral test battery

Behavioral test battery was carried out with male mice (19 *Nlgn3*^*hse*^ mutant mice and 18 littermate WT mice, and 19 *Nlgn3*^*mf*^ mutant mice and 17 littermate WT mice) and started at 9–11 weeks of age. The behavioral test battery included general health and neurological screening, light/dark transition test, open field test, elevated plus maze test, hot plate test, rotarod test, startle response/prepulse inhibition, and Porsolt forced swimming test. All the behavioral testing was performed between 8:30 a.m. and 18:30 p.m. Prior to all experiments, mice were left undisturbed in the testing room for at least 30 min to allow acclimation. After each trial of experiment the apparatus was thoroughly cleaned with hypochlorous water to eliminate any scent to prevent giving a bias as olfactory cue to a next subject. All the behavioral testing except the light/dark test, was performed with illumination level 100 lx. The detailed procedures for each behavioral testing are as follows. After the behavioral test battery, same batches of mice were subjected to Barnes maze test and contextual and cued fear conditioning with 0.3 mA footshock paradigm (please see below).

General health and neurological screening: Health status including body weight, rectal temperature, and neuromuscular strength was examined at the first day of the series of the behavioral testing. Neuromuscular strength was examined by the grip strength test and wire hang test as described [16]. A grip strength meter (O’Hara & Co., Tokyo, Japan) was used to assess forelimb grip strength. Each mouse was tested three times and the greatest value measured was used for statistical analysis. A box (215 × 22 × 23 cm) with a wire mesh grid (10 × 10 cm) on its top (O’Hara & Co., Tokyo, Japan) was used for the wire hang test. Latency to fall was recorded with a 60 s cutoff time.

Light/dark transition test: Light/dark transition test was conducted as previously reported [17]. Mice were placed into the dark chamber and allowed to move freely in the light (380 ± 20 lx) and dark chamber through the opening in between for 10 min. The total number of transitions between chambers, time spent in each side, latency to the first transmission to the light chamber, and distance traveled in each chamber were recorded.

Open field test: Locomotor activity was measured in an open field apparatus (40 × 40 × 30 cm; Accuscan Instruments, Columbus, OH, USA) as described [18]. Mice were placed into left corner of the apparatus and allowed to move freely for 120 min. Total distance traveled, vertical activity (rearing measured by counting the number of photobeam interruptions), time spent in the center (20 × 20 cm) of the open field area, and the stereotypic counts were recorded using VersaMax system (Accuscan Instruments, Columbus, OH, USA).

Elevated pulse maze test: The elevated plus maze test was conducted as described [19]. The elevated plus maze (O’Hara & Co., Tokyo, Japan) consisted of four arms (25 × 5 cm) arranged in plus shape with a 5 × 5 cm square in the center. Two of the arms were closed with 15 cm high walls and other two were open without walls. The closed and open arms were alternately arranged in the plus maze and the maze was elevated to a height of 55 cm above from floor. Each mouse was placed on the center of the maze facing one of the closed arms. Mouse behavior was recorded during a 10 min test period. The time spent in the open and closed arm, the number of entries into the arms, and the distance traveled were recorded.

Hot plate test: The hot plate test was conducted as described [20]. Mice were placed on a 55.0 ± 0.3 °C hot plate (Columbus Instruments, Columbus, OH, USA), and latency to the first front-paw response (rubbing the paws) was recorded.

Porsolt forced swim test: The subject was placed in a cylindrical container (12 cm in diameter) of water (12 cm in depth and 23–24 °C in temperature) in the illuminated chamber (800–850 lx) and allowed to freely move for 10 min. Total distance traveled and percentage of immobility were measured. The same test was repeated on the following day.

Acoustic startle response and prepulse inhibition tests: The acoustic startle responses and prepulse inhibition tests were conducted using startle reflex measurement system (O’Hara & Co., Tokyo, Japan) as described [18]. The subject mouse was put in the test equipment, a Plexiglas cylinder and the cylinder was placed in the test apparatus with background noise level at 70 dB for 10 min for habituation. A test session consisted of 6 trial types: two types for startle stimulus only trials, and four types for prepulse inhibition trials. The duration of white noise that was used as the startle stimulus was 40 ms for all trial types. The startle response was recorded for 140 ms (measuring the response every 1 ms) starting with the onset of the prepulse stimulus. The peak startle amplitude recorded during the 140 ms sampling window was used as the dependent variable. The intensity of startle stimulus was 110 or 120 dB. The prepulse sound was presented 100 ms before the startle stimulus, and its intensity was 74 or 78 dB. Four combinations of prepulse and startle stimuli were employed (74–110. 78–110, 74–120, and 78–120). Six blocks of the 6 trial types were presented in pseudorandom order such that each trial type was presented once within a block. The average intertrial interval was 15 s (range: 10–20 s). The startle amplitude and percentage of prepulse inhibition was measured.

### Five-trial social novelty test

Five-trial social novelty test was carried out with 26 *Nlgn3*^*hse*^ mutant mice and 22 littermate WT mice, and 26 *Nlgn3*^*mf*^ mutant mice and 17 littermate WT mice, essentially according to the previous report [12]. Subject mice (P26-P32) and younger social stimulus mice (WT, P21-P28) were employed in this study. The stimulus mice were marked with a yellow spot on the neck by bleaching under anesthesia to discriminate from the subject mice under the video recording. The experimental arena was a transparent acrylic resin box (30 × 15 × 30 cm), filled with 450 ml of homecage bedding (Paper-clean, Japan SLC, Japan) and illuminated at 100 lx. Subject mice were gently introduced into the box and allowed to acclimate for 15 min. For the first trial, a stimulus mouse (referred to as “Stranger-1”) was introduced into the cage, permitting free interaction between the mice for 4 min. This procedure was replicated for four consecutive trials with 5-min intervals in between, allowing the subject mice to be acquainted with the stimulus mouse. During the fifth trial, a novel mouse (Stranger-2) was introduced instead of the “Stranger-1”. Every subject mouse was temporarily placed in a numbered cage after the test. The subject mice were ear-punched following the completion of all tests. The punched-out samples were stored at − 20 °C and genotyped after manual video analysis. Social interaction behavior was manually scored from TIFF image stacks (4 frame/s) before the genotyping. The social interaction duration included the time for nose-to-nose sniffing, anogenital sniffing, allogrooming, other forms of non-aggressive physical contact, and following the stimulus mouse.

### Social conditioned place preference test

After ear-punching and a week’s rest, mutant and littermate WT mice at P33-P40 (23 *Nlgn3*^*hse*^ mutant mice and 26 littermate WT mice, and 24 *Nlgn3*^*mf*^ mutant mice and 15 littermate WT mice) were subjected to a sCPP test [21]. The apparatus is a 30 × 30 cm clear acrylic resin box, equally divided into two chambers by a central sliding partition having a circular opening, illuminated at 100 lx. Each chamber was filled with 1 cm of different novel bedding (Alpha-dri, Shepherd Specialty Papers, Watertown, TN, USA; Kaytee Soft Granules [discontinued], Petco, Irvine, CA, USA; or Care-feeaz, Hamri Co., Ibaraki-ken, Japan). For the pre-test, mice were gently placed in the apparatus for 30 min of free exploration. Following the pre-test, mice were group-housed in a home cage with “bedding A” for social conditioning over 24 h. Subsequently, the mice were separated and individually housed on "bedding B" for isolated conditioning, for another 24 h. On the third day, the apparatus was set up with both conditioned beddings, and the mice were allowed free exploration for 30 min. The left and right positions of two types of bedding were alternated for each mouse during the tests; however, all mice were consistently introduced from the right side. For each individual, the bedding positions remained the same from the pre-test to the post-test. The bedding used for social and isolated contexts was alternated between each group-housed cage, to ensure the counterbalance. The duration that the subjects spent on the “social context” was recorded and analyzed using an ImageJ plug-in [22].

### Barnes maze test

The Barnes maze test was conducted with mice used for behavioral test battery on a white circular platform, 1.0 m in diameter, with 12 holes equally placed around the perimeter (O’Hara & Co., Tokyo, Japan) elevated 75 cm from the floor as previously described [20]. The platform was illuminated with 1200 lx lighting and one of the 12 holes leads to a black Plexiglas escape box (17 × 13 × 7 cm). The particular hole which leads to the escape box was assigned to each subject as a target. Location of the target hole were evenly assigned to WT and mutant mice. Prior to the test, the subject had habituation trial to become familiar with the maze and the escape box. Each trial test started with emergence of the subject by retracting the wall around the subject at the center of the platform and ended when the subject entered the escape box through their assigned target hole or 5 min elapsed. The latency and distance for the subject to reach the target hole and the number of times that the subject visited incorrect holes were measured in all the tests. Two to three trials per day were conducted for 3 consecutive days. On day 4, the mice received probe trial test that conducted without the escape box for 3 min. During the probe trial test, staying time around the target was measured to confirm that this spatial task was acquired based on navigation by distal environment room cues. Mice were left undisturbed until receiving next probe trials. On day 28, the mice once again received a probe trial test to check remote memory. The dwell time spent wandering around each hole was recorded using Image BM software.

### Radial maze

Radial maze test was carried out with 14 *Nlgn3*^*hse*^ mutant mice and 14 littermate WT mice, and 20 *Nlgn3*^*mf*^ mutant mice and 20 littermate WT mice. The automated eight-arm radial maze apparatus (O’Hara & Co., Tokyo, Japan) was used as previously described [23]. The maze consisted of a regular octagonal platform connecting eight narrow arms (9 × 40 cm) made of white plastic and the walls (25 cm high) made of transparent plastic and illuminated at 100 lx. Program-controlled automatic gates were set between the platform and the arms. At the distal end of each arm, a food pellet well (1.4 cm deep and 1.4 cm in diameter) was set, equipped with a photodetector to automatically record pellet intake. Four distinct cues were hung at the corners of the ceiling. The position of the maze and the direction of the arms remained consistent throughout the experiment. One week before the training phase, the mice were subjected to a food restriction (8 g/day for 4 mice) and were weighed daily to reduce their weight to 80–85% of their initial weight, ensuring they remained hungry without compromising health. From the eighth day, mice explored the maze and consumed scattered pellets for 30 min as a pre-training. Subsequently, they underwent advanced pre-training to take a food pellet from each food well, repeating this process eight times until all arms had been traversed. Once these pre-training trials were completed, the actual maze acquisition trials began. In the spatial working memory task, each of the eight arms contained a food pellet. Initially, the mouse was positioned on the central platform with all gates closed. Each trial began with all gates being simultaneously opened, allowing the subject to explore and consume the food pellets. An "entry" was defined as the entering beyond 5 cm from the platform into a specific arm, resulting in the closure of the others seven gates. Once the mouse returned to the central platform, the remaining gate closed, confining the mouse to the central platform for a 5-s interval. Thereafter, all gates were simultaneously reopened, allowing the mouse to make its next choice. The trial ended when the subject had consumed all the food pellets or when 25 min had elapsed. The mice underwent one trial daily. Two weeks later, once they reached a plateau in their food collection speed and accuracy, the difficulty level increased. The first interval following the consumption of the fourth food pellet was termed “delay-after-4^th^”. The delay-after-4th was sequentially set at 30 s, 120 s, and 300 s, with each duration lasting for two days. All other intervals were kept at 5 s. Metrics including arm choice, time to collect pellets, travel distance, number of different arms chosen within the first eight choices, revisits, and omission errors were automatically recorded.

### Fear conditioning

Contextual fear conditioning using 0.5 mA footshock and fear memory extinction test were conducted with 10 *Nlgn3*^*hse*^ mutant mice and 9 littermate WT mice, and 14 *Nlgn3*^*mf*^ mutant mice and 17 littermate WT mice as previously described[24, 25]. Mice were handled for 1 min per day for 1 week, prior to the experiment. Contextual fear conditioning was conducted in a small conditioning chamber made of transparent plastic surrounded by a sound-attenuating chest (O’Hara & Co., Tokyo, Japan). Mice were placed in the conditioning chamber and two footshocks (0.5 mA, 2 s) were delivered at 58 and 118 s after entry to the chamber. Twenty-four hours later, mice were re-exposed to the conditioned chamber for 30 min without receiving a footshock again for extinction training. The initial 5 min of the extinction session was also referred to as "Test 1", to assess the contextual fear memory. After another twenty-four hours, the mice underwent a 5-min "Test 2" without footshock to evaluate the outcome of the extinction training. Percentage of freezing time was measured using ImageFZ software with image capture rate of 1 frame/s.

Contextual and cued fear conditioning using 0.3 mA footshock were conducted with mice used for behavioral test battery as described previously [24]. The tests consisted of three sessions, conditioning (Day 1), and contextual and cued tests (Day 2 and 30). In the conditioning session, a 55 dB white noise, which served as the conditioned stimulus (CS), was played for 30 s from 120, 240, and 360 s. During the last 2 s of the tone, a footshock of 0.3 mA was delivered as the unconditioned stimulus (US). Each mouse received three CS-US pairings with 2 min interstimulus interval. Contextual testing was conducted 24 h after conditioning (Day 2). The mice were placed in the same chamber that contextual conditioning was taken place and monitored for freezing for 5 min. Cued testing with altered context was conducted 3 h after contextual test in a triangular box (35 × 35 × 40 cm) made of white opaque Plexiglas with illumination level 30 lx. Freezing behavior was assessed during a 3 min free exploration, followed by a 3 min presentation of the tone. Contextual and cued tests were conducted again at Day 30 to assess remote memory. Percentage of freezing time was measured using ImageFZ software with image capture rate of 2 frame/s.

## Results

### Decreased anxiety in ***Nlgn3***^***mf***^ mutant mice

To comprehensively evaluate the effects of impairments in the canonical and noncanonical NLGN3 pathways on behavior, we first subjected the male *Nlgn3*^*hse*^ and *Nlgn3*^*mf*^ mutant mice and their WT littermates to a behavioral test battery (Table 1 and Additional file 1: Table S1). Both *Nlgn3*^*hse*^ and *Nlgn3*^*mf*^ mutants grew normally and their general health and neurological status, including body weight, rectal temperature, wire hang latency, and grip strength of the mutants were comparable to those of WT mice. The hot plate latency and the startle amplitude in the prepulse inhibition test were comparable between both *Nlgn3*^*hse*^ and *Nlgn3*^*mf*^ mutants and control mice, suggesting no obvious changes in the somatosensory system. *Nlgn3*^*hse*^ mutants showed no significant changes in the open field test, light and dark transition test, elevated plus maze test, and Porsolt forced swim test. In contrast, *Nlgn3*^*mf*^ mutants exhibited increased open arm entries and stay time in the open arm in the elevated plus maze test, implying lower anxiety level in the *Nlgn3*^*mf*^ mutant mice.

**Table 1:** Behavioral test battery results of  Nlgn3^*hse*^ and Nlgn3^mf^ mutant mice

Genotypes	WT	Nlgn3hse	P-Value	Statistics	WT	Nlgn3mf	P-Value	Statistics
N =	18	19			17	19		
General health								
Body Weight (g)	26.19 ± 0.45	25.29 ± 0.70	0.293	(1)	25.31 ± 0.59	24.79 ± 0.52	0.515	(5)
Rectal Temperature (°C)	36.86 ± 0.18	36.67 ± 0.16	0.440	(2)	35.11 ± 0.21	35.22 ± 0.24	0.735	(6)
Wire Hang (sec)	8.21 ± 1.49	12.21 ± 4.09	0.375	(3)	10.45 ± 2.04	10.63 ± 3.06	0.963	(7)
Grip Strength (N)	0.84 ± 0.05	0.73 ± 0.05	0.089	(4)	0.52 ± 0.05	0.48 ± 0.04	0.577	(8)
Light and dark								
Stay time (sec)								
Light	138.64 ± 13.13	123.79 ± 10.53	0.381	(9)	145.91 ± 11.11	173.34 ± 16.39	0.185	(13)
Dark	468.94 ± 12.92	483.97 ± 10.78	0.376	(10)	462.59 ± 11.13	434.74 ± 16.02	0.1719	(14)
Transitions	24 ± 2	23 ± 2	0.659	(11)	25 ± 2	27 ± 2	0.6432	(15)
Latency to Enter Light (sec)	85.89 ± 17.76	128.68 ± 23.79	0.162	(12)	96.41 ± 17.32	87.00 ± 27.57	0.7802	(16)
Open field								
Total distance (cm)	11157 ± 859	12311 ± 825	0.339	(17)	10032 ± 593	10028 ± 980	0.998	(21)
Center Time (sec)	1836 ± 248	2073 ± 256	0.510	(18)	1386 ± 204	918 ± 139	0.0612	(22)
Stereotypic Activity	12,344 ± 674	13368 ± 1014	0.412	(19)	11138 ± 790	10007 ± 739	0.303	(23)
Vertical Activity	1658 ± 220	1650 ± 209	0.981	(20)	1189 ± 153	980 ± 110	0.2694	(24)
Elevated Plus Maze								
Number of Entries-Total	43.11 ± 2.46	38.68 ± 1.43	0.124	(25)	47.71 ± 2.55	55.79 ± 2.89	0.046	(28)
Open Arm Entry (%)	24.49 ± 2.42	25.88 ± 2.42	0.688	(26)	25.83 ± 2.73	35.57 ± 3.17	0.028	(29)
Open Arm Stay Time (%)	8.53 ± 1.59	10.41 ± 1.85	0.447	(27)	9.66 ± 1.5	20.49 ± 3.19	0.006	(30)
Hot plate								
Latency (sec)	5.29 ± 0.3	5.79 ± 0.47	0.383	(31)	5.11 ± 0.26	5.45 ± 0.31	0.4079	(32)
Prepulse (PP) inhibition								
N =	16	18			17	19		
Startle Amplitude (SA) 110 dB	1.41 ± 0.18	1.52 ± 0.19	0.698	(33)	1.79 ± 0.17	1.53 ± 0.16	0.2767	(39)
SA 120 dB	1.65 ± 0.27	1.68 ± 0.24	0.929	(34)	1.89 ± 0.2	1.76 ± 0.20	0.6552	(40)
PPSoundlevel (PPS) 74 dB–SA110dB	27.58 ± 4.95	41.13 ± 6.22	0.104	(35)	30.85 ± 7.07	32.14 ± 5.66	0.887	(41)
PPS74dB–SA120dB	14.56 ± 6.74	22.64 ± 8.12	0.456	(36)	25.13 ± 7.45	31.34 ± 6.23	0.5241	(42)
PPS78dB–SA110dB	47.7 ± 3.86	56.01 ± 5.41	0.231	(37)	48.01 ± 6.06	48.20 ± 5.67	0.9821	(43)
PPS78dB–SA120dB	37.5 ± 5.56	48.77 ± 6.6	0.207	(38)	41.23 ± 5.87	454.42 ± 5.96	0.707	(44)
Porsolt forced swim								
N =	18	19			17	19		
Immobility (%) Day1	48.91 ± 2.41	45.03 ± 2.50	0.273	(45)	46.468 ± 2.57	46.30 ± 1.94	0.962	(49)
Immobility (%) Day2	58.32 ± 4.30	57.01 ± 2.61	0.793	(46)	60.91 ± 2.44	59.86 ± 1.63	0.752	(50)
Total distance (cm) Day1	733.82 ± 38.62	732.38 ± 31.41	0.977	(47)	722.50 ± 30.67	704.19 ± 25.87	0.664	(51)
Total distance (cm) Day2	541.68 ± 104.43	474.42 ± 23.57	0.524	(48)	418.65 ± 25.80	449.48 ± 21.86	0.388	(52)

### Redundancy of NLGN3-NRXNs and NLGN3-PTPδ pathways in the development of social recognition

**Fig. 1:**
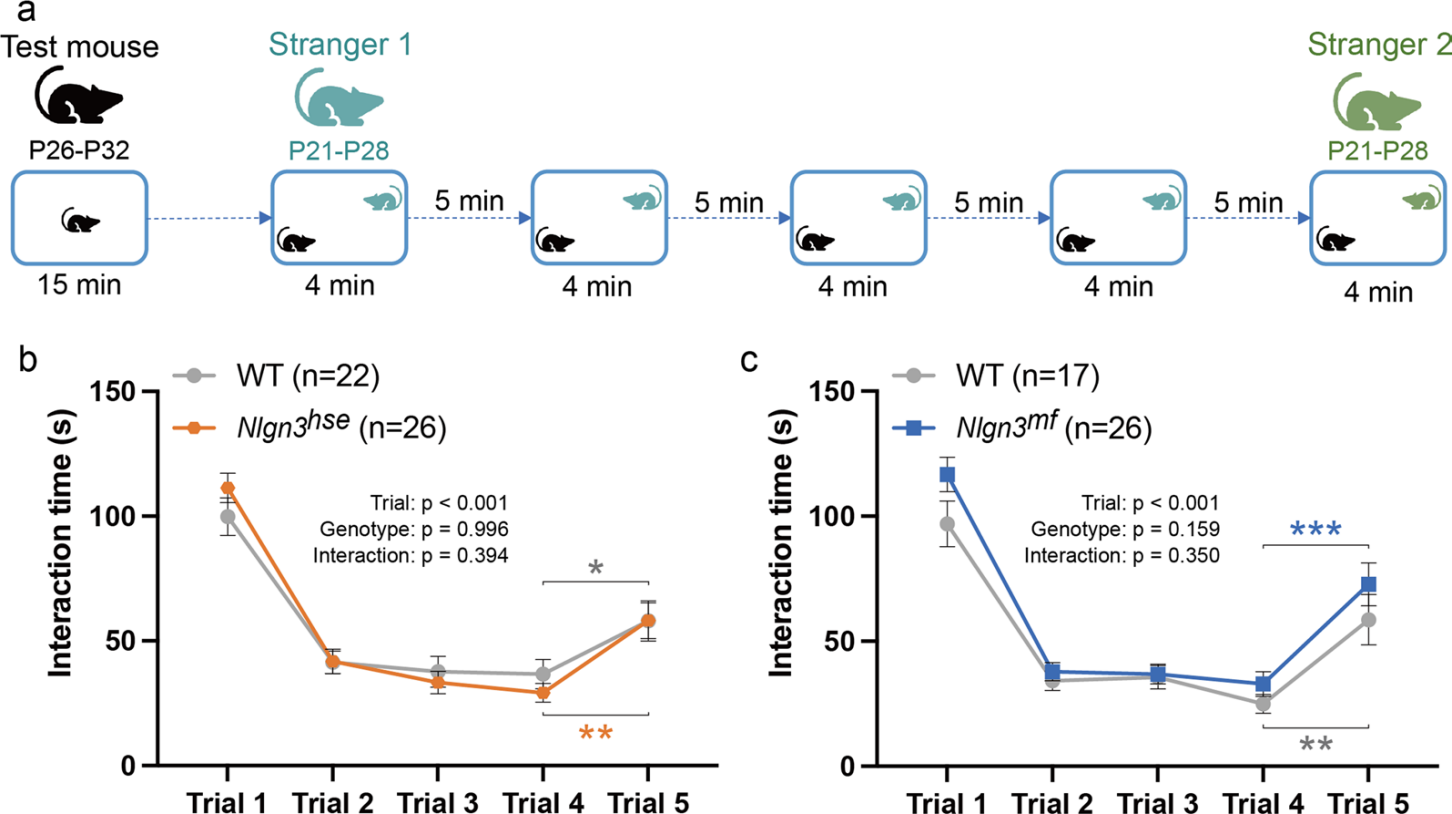
Disruption of either NLGN3-NRXNs or NLGN3-PTPδ pathway has little effect on social novelty recognition. **a** Experimental time-course for the five-trial social novelty test. **b** Mean social interaction time in wild-type (WT) and *Nlgn3*^*hse*^ mutant mice. **c** Mean social interaction time in WT and *Nlgn3*^*mf*^ mutant mice. Data are presented as mean ± SEM. *P < 0.05, **P < 0.001, and ***P < 0.0001, Paired two-tailed t-test

Since a previous study demonstrated that a NLGN3 KO impaired social novelty recognition in juvenile mice [12], we aimed to identify the pathway specifically responsible for the development of social recognition. For this purpose, we employed the five-trial social recognition test, which evaluates habituation to a stranger mouse during the first four trials, and novelty response to a novel stranger on the fifth trial (Fig. 1a) [26]. We observed that both *Nlgn3*^*hse*^ and *Nlgn3*^*mf*^ mutants as well as their littermate WT mice at P26–P32 spent a significant amount of time sniffing and chasing unfamiliar mice upon their initial encounter (Fig. 1b, c). However, after the second encounter, both *Nlgn3*^*hse*^ and *Nlgn3*^*mf*^ mutants as well as their littermate control mice spent only a minimal amount of time interacting with the stimulus mice, maintaining a consistently low level of interaction until the stimulus mice were replaced by a new unfamiliar mouse, "Stranger 2", in the fifth trial. Both *Nlgn3*^*hse*^ and *Nlgn3*^*mf*^ mutants as well as littermate control mice spent significantly more time in the fifth trial interacting with the "Stanger 2" than in the fourth trial interacting with the “Stranger 1” (Paired t-test: P = 0.0002 and P = 0.0198, for the *Nlgn3*^*hse*^ mutants and their littermate WT mice, respectively; P < 0.0001 and P = 0.0026, for the *Nlgn3*^*mf*^ mutants and their littermate WT mice, respectively) ( Fig. 1b, c), indicating that both the *Nlgn3*^*hse*^ and *Nlgn3*^*mf*^ mutant mice could distinguish Stranger 2 from Stranger 1 as two distinct individuals. These results suggest that lack of either the canonical NLGN3-NRXNs or noncanonical NLGN3-PTPδ pathway has negligible effects on the development of social recognition. Considering that *Nlgn3* KO mice showed impaired social recognition in the five-trial test [11], the canonical and noncanonical NLGN3 pathways may play redundant roles in the development of social recognition.

### The noncanonical NLGN3 pathway is required for social conditioned place preference

**Fig. 2:**
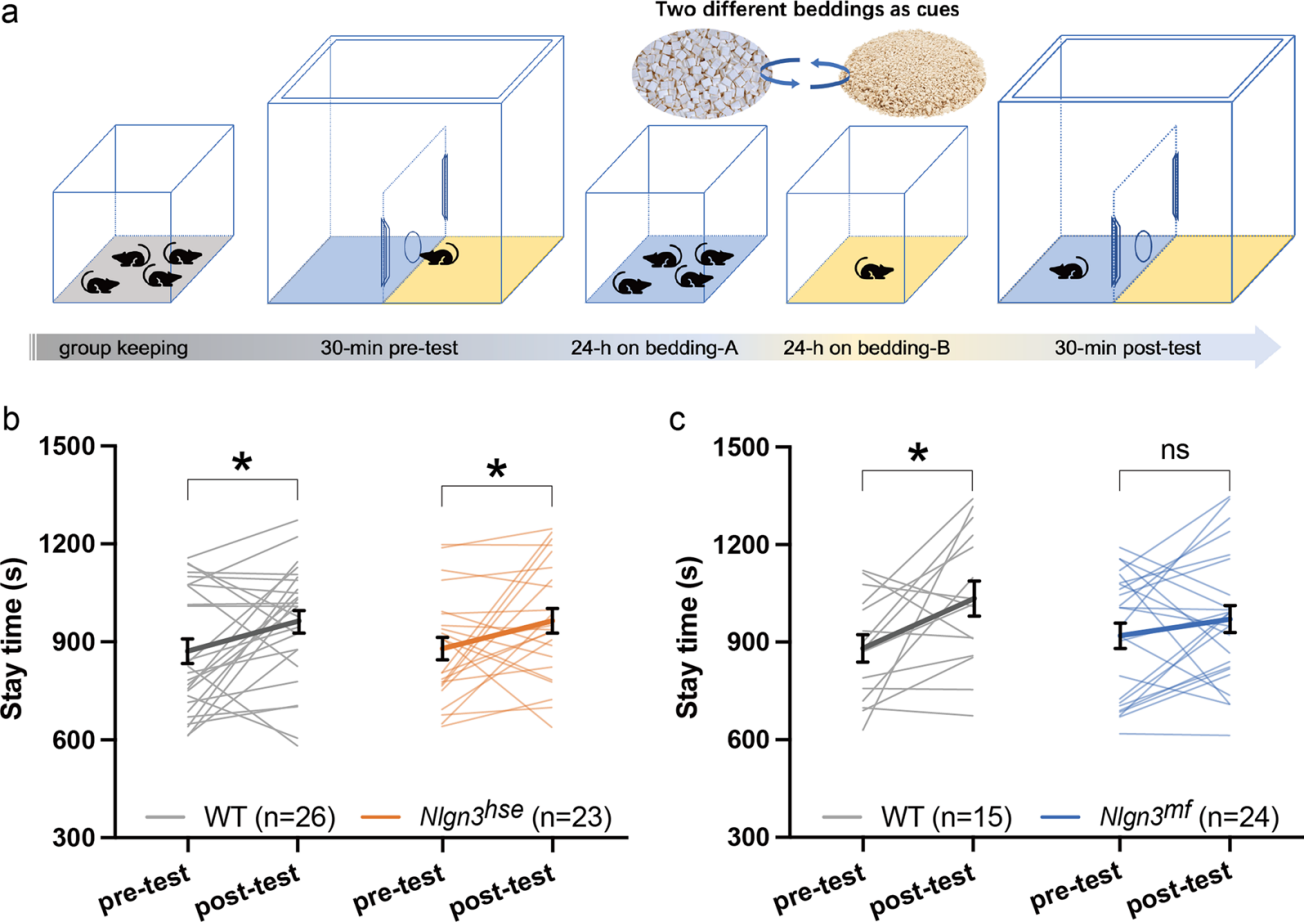
Impaired social conditioned place preference in *Nlgn3*^*mf*^ mutant mice. **a** Schematics of the sCPP test. **b** Scatter plot of time spent in the "social" chamber during the pre- and post-test for WT and *Nlgn3*^*hse*^ mutant mice. There was no significant difference between WT and *Nlgn3*^*hse*^ mutant mice (RM two-way ANOVA: time effect, F (1, 94) = 6.040, P = 0.0158; genotype effect, F (1, 94) = 0.01403, P = 0.9060; time × genotype interaction, F (1, 94) = 0.01029, P = 0.9194). **c** Scatter plot of time spent in the "social" chamber during the pre- and post-test for WT and *Nlgn3*^*mf*^ mutant mice. There was no significant difference between WT and *Nlgn3*^*mf*^ mutant mice (RM two-way ANOVA: time effect, F (1, 74) = 5.129, P = 0.0265; genotype effect, F (1, 74) = 0.1167, P = 0.7336; time × genotype interaction, F (1, 74) = 1.272, P = 0.2630). Summarized data are presented as mean ± SEM. *P < 0.05, Paired two-tailed t-test

Juvenile *Nlgn3* KO mice show impaired sCPP, suggesting a defect in social reward processing [11]. To determine the NLGN3 pathway responsible for the social reward behavior, we subjected *Nlgn3*^*hse*^ and *Nlgn3*^*mf*^ mutant mice and their littermate WT control mice to the sCPP test. In this test, juvenile mice were conditioned with familiar littermates [27]. In the sCPP paradigm, test mice were group-housed on bedding-A and then individually housed on bedding-B to condition social and solitary environments, respectively (Fig. 2a). Both *Nlgn3*^*hse*^ mutant and littermate WT mice exhibited sCPP as indicated by a longer time spent in the social cue chamber in the post-test than in the pre-test (Paired t-test: P = 0.0485 and P = 0.0288, for *Nlgn3*^*hse*^ mutants and their littermate WT mice, respectively) (Fig. 2b). In contrast, the time spent in the social cue chamber in the pre- and post-test was comparable for *Nlgn3*^*mf*^ mutant mice, suggesting no obvious sCPP (Paired t-test, P = 0.2259) (Fig. 2c). In contrast, the littermate control mice for *Nlgn3*^*mf*^ mutants, exhibited a robust sCPP (Paired t-test, P = 0.0256). These results suggest that the noncanonical NLGN3-PTPδ pathway, but not the canonical pathway, specifically contributes to social reward processing.

### Remote spatial reference memory is impaired in ***Nlgn3***^***mf***^ mutant mice

**Fig. 3:**
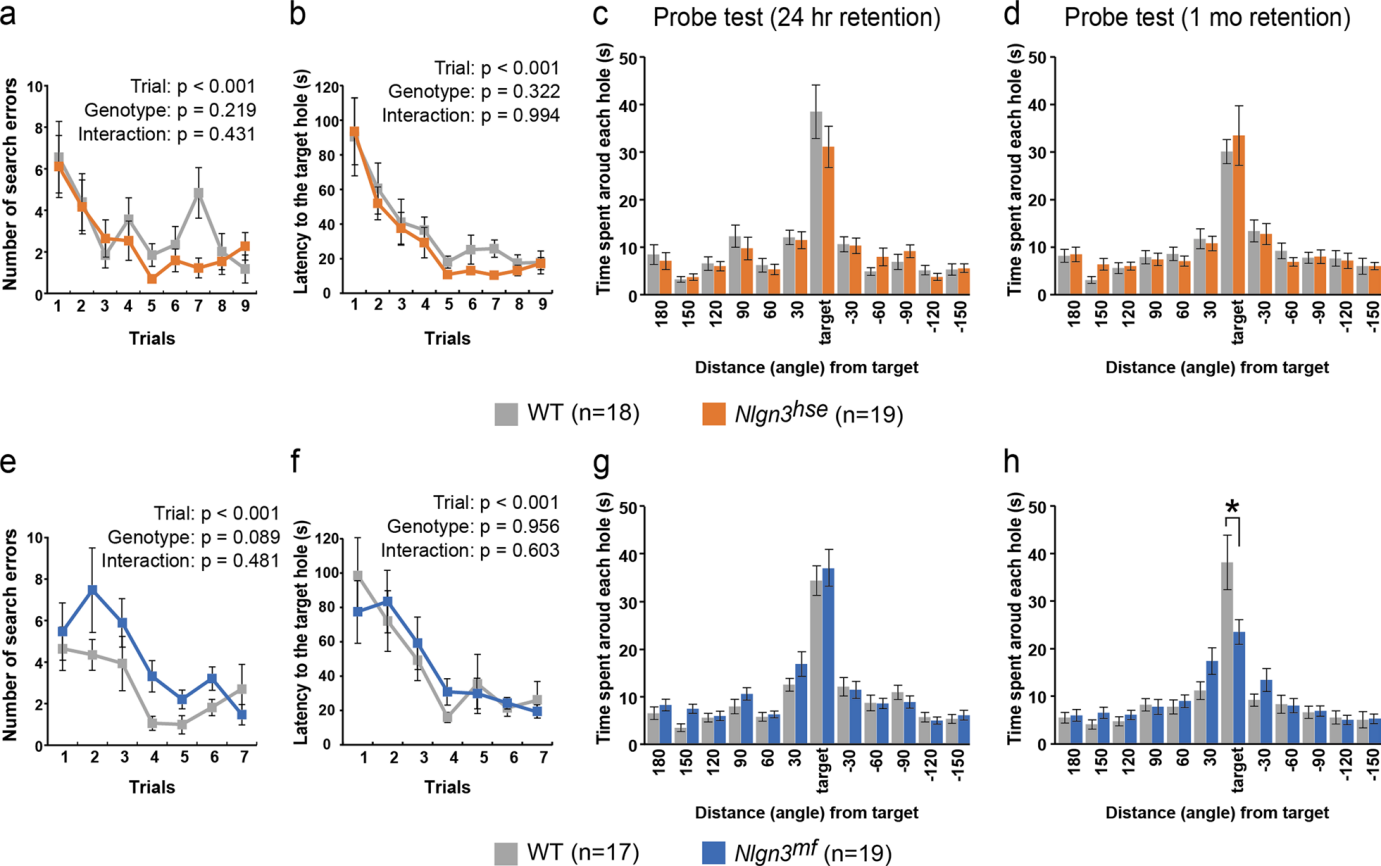
Impaired remote spatial reference memory in *Nlgn3*^*mf*^ mutant mice. Spatial reference memory of the *Nlgn3*^*hse*^ mutant and littermate WT mice (**a**–**d**), and that of the *Nlgn3*^*mf*^ mutant and littermate WT mice (**e**–**h**) were examined using the Barnes maze test. **a**, **e** Number of errors before reaching the target hole across training. **b**, **f** Latency to reach the target hole across training. There was no significant difference between WT and *Nlgn3*^*hse*^ mutant mice, and between WT and *Nlgn3*^*mf*^ mutant mice during training session. Summary statistics are shown in each panel. **c**, **d**, **g**, **h**, Time spent around each hole in the probe trial conducted 24 h (**c**, **g**) and 1 month (**d**, **h**) after last training session. All values are presented as the mean ± SEM. *P < 0.05, Two-tailed t-test

We next examined the cognitive function of these *Nlgn3* mutant mice using different types of learning and memory tasks. These mutants were first subjected to the Barnes maze to assess their spatial learning and memory. Both *Nlgn3*^*hse*^ and *Nlgn3*^*mf*^ mutants and their littermate WT mice learned to locate the target hole during the training period, as indicated by gradual reductions in the number of search errors and escape latency (Fig. 3a, b, e, f). To evaluate recent and remote memory, we conducted the first probe test and second probe test at one day and one month after the last day of training, respectively. In both probe tests, both the *Nlgn3*^*hse*^ mutant and littermate WT mice moved to the hole where the escape box had been and the time spent around the target hole was comparable between genotypes (t-test: P = 0.303 and 0.632 for the first and second probe test, respectively) (Fig. 3c, d), suggesting a negligible effect of impairment in the canonical pathway on the acquisition and retention of spatial reference memory. In contrast, the *Nlgn3*^*mf*^ mutants spent significantly less time around the target hole than littermate WT mice in the second probe test, despite both the *Nlgn3*^*mf*^ mutant and WT mice spent comparable time around the target in the first probe test (t-test: P = 0.615 and 0.031 for first and second probe test, respectively) (Fig. 3g, h). These results suggest that the noncanonical NLGN3-PTPδ pathway selectively contributes to the consolidation of remote spatial reference memory.

### Spatial working memory is unaltered in ***Nlgn3***^***hse***^ and ***Nlgn3***^***mf***^ mutant mice

**Fig. 4:**
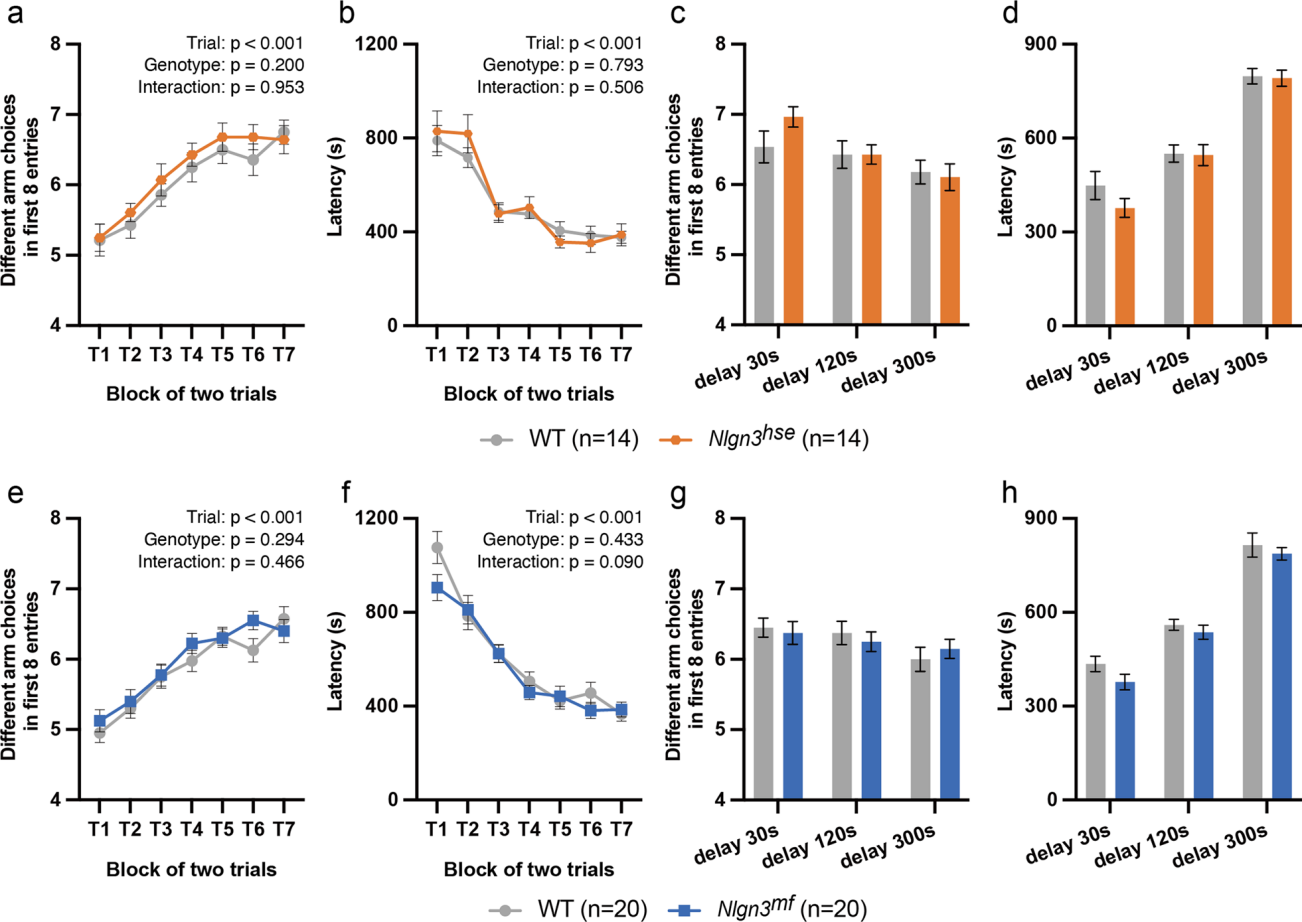
Little effect on spatial working memory by disruption of either NLGN3-NRXNs or NLGN3-PTPδ pathway. Spatial working memory of the *Nlgn3*^*hse*^ mutant and littermate WT mice (**a**–**d**), and that of the *Nlgn3*^*mf*^ mutant and littermate WT mice (**e**–**h**) were examined by eight-arm radial maze test. **a**, **c**, **e**, **g**, Number of different arm choices among the first 8 entries. **b**, **d**, **f**, **h** Latency to take all pellets. A delay was applied after the first 4 pellets were consumed (**c**, **d**, **g**, **h**). All values are presented as the mean ± SEM. There was no significant deference in different arm choices and latency to take all pellets between WT and *Nlgn3*^*hse*^ mutant mice, and between WT and *Nlgn3*^*mf*^ mutant mice. Summary statistics are shown in each panel

We next examined the spatial working memory with the eight-arm radial maze test. The test was performed with food-restricted mice, using food pellets as a reward. We observed no significant differences between the *Nlgn3*^*hse*^ mutant and littermate WT mice, and between the *Nlgn3*^*mf*^ mutant and littermate WT mice in the number of different arm choices among the first eight entries and the latency to take all food pellets (Fig. 4a, b, e, f). After mice achieved a plateau in both speed and accuracy in acquiring rewards, we introduced a one-off delay (30 s, 120 s, or 300 s) after the fourth pellet was taken. The number of different arm choices among the first eight entries and total time required to obtain all rewards was again comparable between both mutants and their littermate WT mice (Fig. 4c, d, g, h). These results suggest that the lack of either the canonical or noncanonical NLGN3 pathway has little effect on spatial working memory.

### Differential effects of an impaired canonical or noncanonical NLGN3 pathway on fear conditioning and extinction

**Fig. 5:**
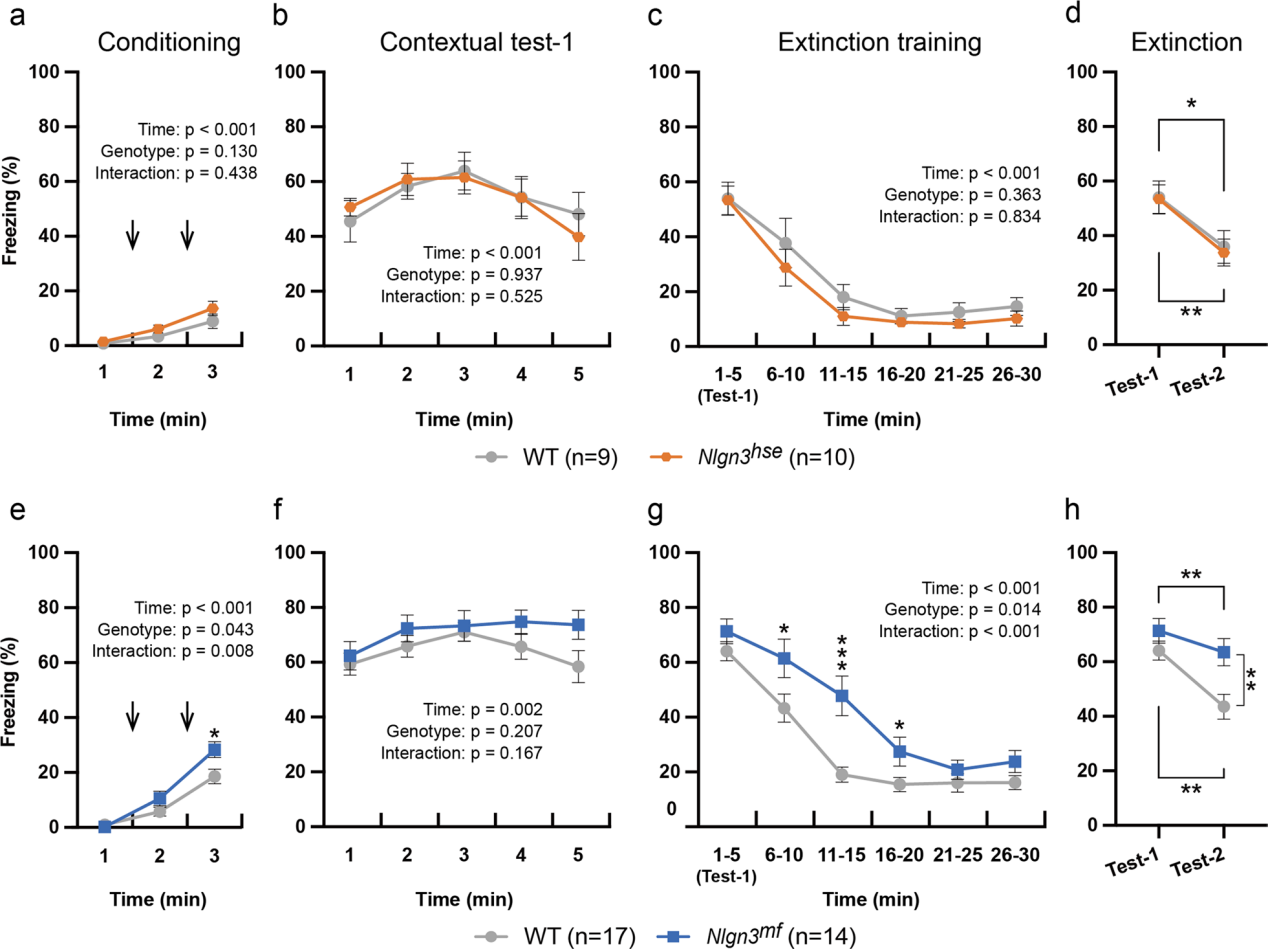
Enhanced freezing responses of *Nlgn3*^*mf*^ mutant mice in the contextual fear conditioning with a 0.5 mA electric shock. Freezing responses during conditioning with footshocks of 0.5 mA (**a**, **e**), contextual test-1 (**b**, **f**), extinction session (**c**, **g**), and contextual-test 2 (**d**, **h**) of the *Nlgn3*^*hse*^ mutant mice and their littermate WT mice (**a**–**d**) and of the *Nlgn3*^*mf*^ mutant mice and their littermate WT mice (**e**–**h**) are quantified. Arrows represent footshock. There were no significant differences in freezing responses between the *Nlgn3*^*hse*^ mutant mice and their littermate WT mice, while the *Nlgn3*^*mf*^ mutant mice exhibited significantly higher freezing responses than their littermate WT mice during conditioning, extinction session, and contextual-test 2. Summary statistics are shown in each panel. All values are presented as the mean ± SEM. *P < 0.05, **P < 0.01, and ***P < 0.001, Two-tailed t-test in **e**, **g**, **h**, and paired two-tailed t-test in **d**, **h**

Because, in the behavioral test battery, both *Nlgn3*^*hse*^ and *Nlgn3*^*mf*^ mutant mice showed unaltered pain sensitivity and startle responses, and no obvious increment in fear and anxiety, though *Nlgn3*^*mf*^ mutant mice rather exhibited decreased anxiety, we next conducted contextual and cued fear conditioning with footshocks as unconditioned stimuli to evaluate fear learning and memory of these mutant mice. *Nlgn3*^*hse*^ and *Nlgn3*^*mf*^ mutant mice and their littermate WT mice were subjected to a contextual fear conditioning and extinction paradigm. During the conditioning period, the freezing behavior observed before the first presentation of a 0.5 mA electrical footshock for 2 s was minimal and not different between the *Nlgn3*^*hse*^ mutant and littermate WT mice. After footshocks, the freezing responses of the *Nlgn3*^*hse*^ mutant and littermate WT mice similarly increased (Fig. 5a). In contrast, *Nlgn3*^*mf*^ mutant mice showed a higher freezing level than the littermate WT mice after footshocks (t-test: P = 0.019) (Fig. 5e), suggesting enhanced fear conditioning. One day after conditioning, mice were placed in the shock chamber for 30 min without shock. There was no significant difference in the freezing levels in the contextual test for the first 5 min (Test 1) between the two types of mutants and their respective littermate WT mice (Fig. 5b, f). During the following extinction session, the freezing levels similarly and gradually decreased in the *Nlgn3*^*hse*^ mutant and littermate WT mice (Fig. 5c). In contrast, the freezing level decreased more slowly in *Nlgn3*^*mf*^ mutant mice than in littermate WT mice (Fig. 5g). One day after the extinction session, mice were subjected to a contextual test for 5 min (Test 2) to evaluate extinction. Figure 5d and h show the average freezing rates during Test 1 and Test 2. *Nlgn3*^*hse*^ mutant and littermate WT mice exhibited a clear extinction of fear memory as indicated by a significant decrease in the freezing level from Test 1 to Test 2 (Paired t-test, P = 0.0023 and 0.0197 for WT and *Nlgn3*^*hse*^ mutant mice, respectively) (Fig. 5d). The freezing levels of the *Nlgn3*^*mf*^ mutant and littermate WT mice in the Test 2 were less than those in the Test 1 (Paired t-test, P = 0.0013 and 0.007 for WT and *Nlgn3*^*mf*^ mutant mice, respectively) (Fig. 5h). However, the freezing level in the Test 2 was significantly higher in *Nlgn3*^*mf*^ mutants than in littermate WT mice (t-test, P = 0.0063) (Fig. 5h). These results suggest that lack of the noncanonical NLGN3 pathway enhances acquisition and suppresses extinction of contextual fear memory.

*Nlgn3* KO mice have been reported to show a decreased freezing response in contextual fear conditioning and cued fear conditioning when mice are trained with a paired conditioned stimulus (CS) of 85-dB tone and an unconditioned stimulus (US) of a 0.4 mA footshock [13]. Accordingly, we employed a milder training protocol, pairing a CS of 55-dB white noise with a US of 0.3 mA footshock [24]. Higher freezing levels in this 0.3 mA footshock paradigm than in 0.5 mA footshock paradigm (Fig. 5a, e) during conditioning is probably ascribed to image capture rate (Please see “Materials and methods”).

**Fig. 6:**
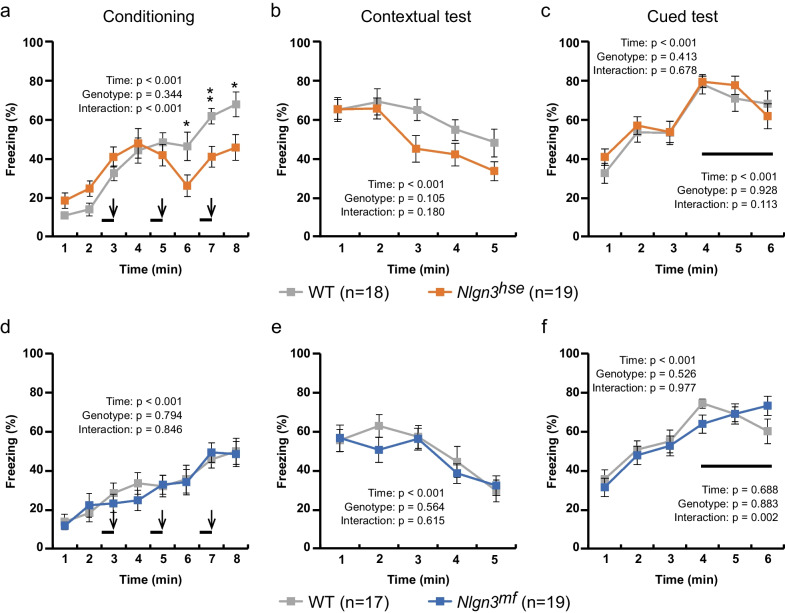
Reduced freezing levels of *Nlgn3*^*hse*^ mutant mice in the contextual fear conditioning with a 0.3 mA electric shock. Freezing responses during conditioning with footshocks of 0.3 mA (**a**, **d**), contextual test (**b**, **e**), and cued test (**c**, **f**) of the *Nlgn3*^*hse*^ mutant mice and their littermate WT mice (**a**–**c**) and of the *Nlgn3*^*mf*^ mutant mice and their littermate WT mice (**d**–**f**) are quantified. Bold lines and arrows represent tone and footshock, respectively. The *Nlgn3*^*hse*^ mutant mice showed significantly less freezing responses than their littermate WT mice during conditioning, while no significant difference was detected between the *Nlgn3*^*mf*^ mutant mice and their WT littermate. Summary statistics are shown in each panel. All values are presented as the mean ± SEM. *P < 0.05 and **P < 0.01, Two-tailed t-test in **a**

During the conditioning session, the freezing levels of *Nlgn3*^*hse*^ mutant mice did not increase as efficiently as that of their WT littermates and the freezing levels after the second and third footshocks were significantly lower than those observed in littermate WT mice (t-test, P = 0.03, 0.004, and 0.022 for 5–6, 6–7, and 7–8 min, respectively) (Fig. 6a). In line with this result, the freezing levels during the 5 min contextual test conducted one day after conditioning tended to be lower in *Nlgn3*^*hse*^ mutant mice than in their WT littermates (Fig. 6b). In contrast, in the cued test, there was no difference in freezing levels between *Nlgn3*^*hse*^ mutant mice and their WT littermates (Fig. 6c). These results suggest that the canonical NLGN3 pathway may contribute to the acquisition of contextual fear memory with a weak US. In contrast, there were no significant differences in freezing levels between *Nlgn3*^*mf*^ mutant mice and their WT littermates in conditioning, contextual testing, or cued testing sessions (Fig. 6d-f). We further conducted contextual and cued tests 30 days after the conditioning to evaluate remote fear memory (Additional file 2: Fig. S1). There were no significant differences in freezing levels between *Nlgn3*^*hse*^ mutant mice and their WT littermates, and between *Nlgn3*^*mf*^ mutant mice and their littermate WT mice, suggesting that the disruption of either canonical or noncanonical pathway has little effect on remote fear memory formation.

## Discussion

In this study, we demonstrated that the noncanonical NLGN3-PTPδ pathway and the canonical NLGN3-NRXN pathway play distinct roles in higher order brain functions by utilizing two *Nlgn3* mutant mouse lines, *Nlgn3*^*hse*^ and *Nlgn3*^*mf*^, which selectively lack the canonical and noncanonical pathways, respectively. A comprehensive behavioral test battery performed to systematically profile the general developmental status, including such as body weight, muscle strength, pain perception, reflexes, and anxiety levels, indicated no significant abnormalities in *Nlgn3*^*hse*^ or *Nlgn3*^*mf*^ mutant mice except for a slight decrease in anxiety in *Nlgn3*^*mf*^ mutant mice. On the other hand, changes in the social reward system, remote spatial reference memory formation, and fear memory acquisition and extinction were observed in one or both of these mutants, suggesting the involvement of NLGN3 in higher order brain functions. These results appear reasonable considering that *NLGN3* mutations are implicated in ASD and ID.

*Nlgn3* KO mice failed to exhibit novelty toward the second stranger mouse in the five-trial social recognition test and showed no sCPP at juvenile stage [12], and exhibited a defect in social memory in adulthood [11, 13], suggesting that NLGN3 plays an indispensable role in the circuits responsible for social novelty recognition and social reward or motivation processing. Therefore, we expected that one or both of the pathways would be involved in these social behaviors. Contrary to our expectations, both *Nlgn3*^*hse*^ and *Nlgn3*^*mf*^ mutant mice still exhibited novelty toward the second stranger mouse. These results suggest that canonical NLGN3-NRXNs and noncanonical NLGN3-PTPδ pathway play a redundant role in the development of neural circuits required for social novelty recognition and the preservation of one pathway is sufficient, although we cannot exclude the possibility that other as-yet-unidentified NLGN3-related pathways may be involved. A deficit in conspecific recognition observed in *Nlgn3* KO mice is assumed to be related to an olfactory deficiency [13], therefore the olfactory system in *Nlgn3*^*hse*^ and *Nlgn3*^*mf*^ mutant mice seems unaffected.

In contrast, NLGN3-mediated regulation of social reward or motivation seems to specifically require the noncanonical pathway because *Nlgn3*^*mf*^ mutant mice, but not *Nlgn3*^*hse*^ mutant mice, failed to exhibit a clear sCPP. These results indicate that the reward derived from social interactions in *Nlgn3*^*mf*^ mutants is insufficient to substantially affect their behavioral patterns. This is in contrast to the observation that sucrose food pellets effectively changed the foraging strategy of *Nlgn3*^*mf*^ mutant mice in the eight-arm radial maze, indicating an unaltered general reward system and downstream behavioral circuits in *Nlgn3*^*mf*^ mutant mice. Part of the social neural network intersects with the general reward system [28, 29]. Observations from the sCPP and radial maze paradigms suggest that the noncanonical pathway may play an indispensable role within the social reward circuit, but independent of the general reward system. The altered social reward system observed in *Nlgn3*^*mf*^ mutant mice at juvenile stages may contribute to the lack of social preference observed in adulthood [15].

In the Barnes maze, *Nlgn3*^*mf*^ mutant mice failed to reach and stay around the target hole in the second probe test at one month after training, but not in the first probe test at one day after training, indicating selective impairment in the consolidation of remote spatial reference memory. The memory consolidation process is supposed to involve the transfer of memory traces from the hippocampus to the cerebral cortex [30]. Our finding raises the intriguing possibility that formation of new synapses mediated by synaptic organizers such as NLGN3 underlies this memory consolidation process. In fact, selective impairments in remote fear memory have also been observed in mice deficient in IL1RAPL1, one of the postsynaptic organizers interacting with PTPδ [31].

In the contextual fear conditioning paradigm with 0.5 mA footshocks, *Nlgn3*^*mf*^ mutant mice exhibited enhanced freezing during conditioning and extinction session. In contrast, in the contextual and cued fear conditioning with 0.3 mA footshocks, *Nlgn3*^*hse*^ mutant mice displayed decreased freezing during conditioning session. These results suggest that canonical and noncanonical pathways may play opposite roles in the acquisition of fear memory, although the possibility that the canonical and noncanonical pathways may regulate expression of freezing behavior itself rather than fear learning and memory cannot be excluded. We previously found that the balance in excitatory/inhibitory (E/I) synapses in *Nlgn3*^*mf*^ mutant mice is disrupted in the medial prefrontal cortex (mPFC) [15], a brain region which plays a pivotal role in the extinction of fear memory [32]. Thus, the impaired extinction of fear memory observed in *Nlgn3*^*mf*^ mutant mice may be ascribed to the E/I imbalance in the mPFC. In fear conditioning, the circuits involved in memory acquisition are thought to differ depending on the intensity of the unconditioned stimulus [33, 34], which may cause the different phenotypes of these mutants depending on the intensity of footshock.

In the conventional knowledge framework, the “NLGN3 function” is equated with the “function of the NLGN3-NRXN pathway.” Consequently, a NLGN3 KO is perceived as a model of NLGN3-NRXN pathway disruption. This conflation may mislead behavioral studies and misattribute the effects of simultaneous blockade of the noncanonical NLGN3-PTPδ pathway to the consequences of NLGN3-NRXN pathway disruption. A series of behavioral analyses of *Nlgn3*^*hse*^ and *Nlgn3*^*mf*^ mutant mice clearly demonstrated the differential contributions of the canonical and noncanonical NLGN3 pathways to social and learning and memory performance. Interestingly, *Nlgn3*^*mf*^ mutant mice lacking the noncanonical NLGN3-PTPδ pathway were more prone to exhibit memory and social phenotypes compared to *Nlgn3*^*hse*^ mutant mice lacking the canonical NLGN3-NRXN pathway. This may be due to the binding specificities of NLGN3 to PTPδ and NRXNs. *Nlgn* and *Nrxn* family are widely expressed in the brain in an overlapping manner [35, 36] and all NLGN family proteins, including NLGN3, bind to all NRXN family proteins [37]. This redundant binding system may contribute to compromise behavioral phenotypes in *Nlgn3*^*hse*^ mutant mice. Moreover, increment of NLGN3-PTPδ synaptogenic complex is observed in the *Nlgn3*^*hse*^ mutant mouse brain, suggesting that the lack of the canonical pathway causes circuit-level changes [15]. Nevertheless, wide expression in the brain and robust synaptogenic activities of NLGNs and NRXNs [38, 39] suggest a considerably important role in the physiological condition. In contrast, among the leucocyte antigen related family proteins, NLGN3 binds only to PTPδ, and PTPδ binds only to NLGN3 among all NLGN family proteins, implying no compensatory interactions for the noncanonical pathway [15].

In this current study, we dissected out the contributions of the canonical and noncanonical NLGN3 synaptogenic pathways to the regulation of higher order brain functions related to ASD and ID. In the future, dissecting the neural circuit and synapse-level functions of these two pathways will help elucidating the pathogenic mechanisms of ASD and ID associated with *NLGN3* mutations.

### Supplementary Information

**Additional file 1:** **Table S1. **Statistics of behavioral test battery.

**Additional file 2:** **Figure S1.** Unaltered remote fear memory of *Nlgn3*^*hse*^ and *Nlgn3*^*mf*^ mutant mice. Freezing responses during contextual test (**a**, **c**), and cued test (**b**, **d**) of the *Nlgn3*^*hse*^ mutant mice and their littermate WT mice (**a**, **b**) and of the *Nlgn3*^*mf*^ mutant mice and their littermate WT mice (**c**, **d**) 30 days after conditioning with pairing of 55-dB CS and 0.3 mA footshock US are quantified. Bold lines represent tone. Summary statistics are shown in each panel. All values are presented as the mean ± SEM.

## Data Availability

The datasets supporting the conclusion of this study are included in this article.

## Abbreviations

NLGN: Neuroligin
NRXN: Neurexin
PTPδ: Protein tyrosine phosphatase δ
ASD: Autism spectrum disorders
ID: Intellectual disability
sCPP: Social conditioned place preference
CS: Conditioned stimulus
US: Unconditioned stimulus
