# Supplementary figures and images for: Differential contribution of canonical and noncanonical NLGN3 pathways to early social development and memory performance
Source: Mol Brain. 2024 Mar 12;17:16. doi: 10.1186/s13041-024-01087-5 (PMC10935922; doi:10.1186/s13041-024-01087-5)

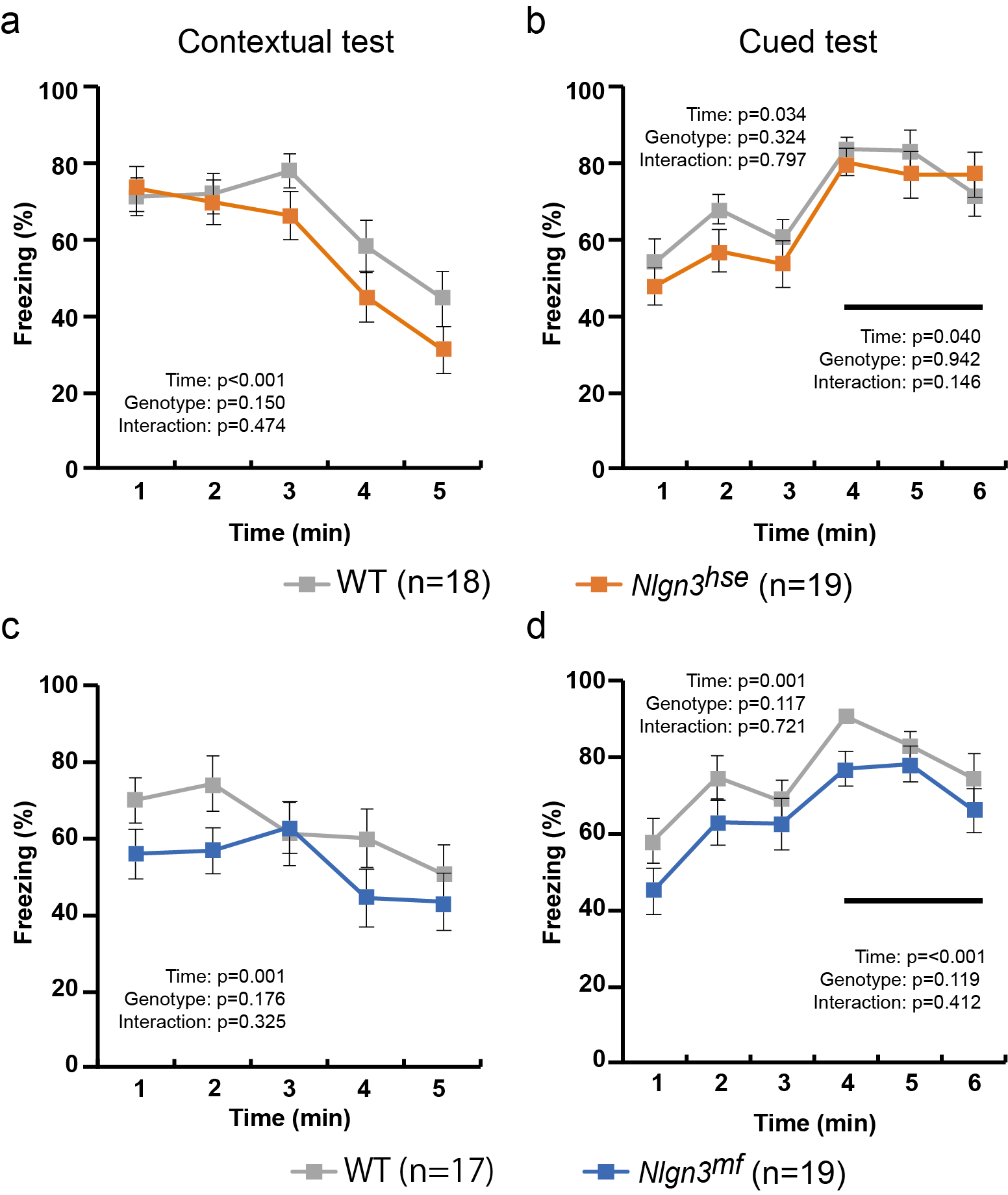

Supplement: Supplementary file 2 — Additional file 2: Figure S1. Unaltered remote fear memory of Nlgn3hse and Nlgn3mf mutant mice. Freezing responses during contextual test (a, c), and cued test (b, d) of the Nlgn3hse mutant mice and their littermate WT mice (a, b) and of the Nlgn3mf mutant mice and their littermate WT mice (c, d) 30 days after conditioning with pairing of 55-dB CS and 0.3 mA footshock US are quantified. Bold lines represent tone. Summary statistics are shown in each panel. All values are presented as the mean ± SEM. [file 13041_2024_1087_MOESM2_ESM.png]
